# Understanding Communication in an Online Cancer Forum: Content Analysis Study

**DOI:** 10.2196/29555

**Published:** 2021-09-07

**Authors:** Anietie Andy, Uduak Andy

**Affiliations:** 1 Penn Medicine Center for Digital Health University of Pennsylvania Philadelphia, PA United States; 2 Division of Urogynecology and Pelvic Reconstructive Surgery Department of Obstetrics and Gynecology Hospital of the University of Pennsylvania Philadelphia, PA United States

**Keywords:** Cancer, Reddit, online forum, natural language processing, latent Dirichlet allocation, Linguistic Inquiry and Word Count, psycholinguistics, social media

## Abstract

**Background:**

Cancer affects individuals, their family members, and friends, and increasingly, some of these individuals are turning to online cancer forums to express their thoughts/feelings and seek support such as asking cancer-related questions. The thoughts/feelings expressed and the support needed from these online forums may differ depending on if (1) an individual has or had cancer or (2) an individual is a family member or friend of an individual who has or had cancer; the language used in posts in these forums may reflect these differences.

**Objective:**

Using natural language processing methods, we aim to determine the differences in the support needs and concerns expressed in posts published on an online cancer forum by (1) users who self-declare to have or had cancer compared with (2) users who self-declare to be family members or friends of individuals with or that had cancer.

**Methods:**

Using latent Dirichlet allocation (LDA), which is a natural language processing algorithm and Linguistic Inquiry and Word Count (LIWC), a psycholinguistic dictionary, we analyzed posts published on an online cancer forum with the aim to delineate the language features associated with users in these different groups.

**Results:**

Users who self-declare to have or had cancer were more likely to post about LDA topics related to hospital visits (Cohen *d*=0.671) and use words associated with LIWC categories related to health (Cohen *d*=0.635) and anxiety (Cohen *d*=0.126). By contrast, users who declared to be family members or friends tend to post about LDA topics related to losing a family member (Cohen *d*=0.702) and LIWC categories focusing on the past (Cohen *d*=0.465) and death (Cohen *d*=0.181) were more associated with these users.

**Conclusions:**

Using LDA and LIWC, we show that there are differences in the support needs and concerns expressed in posts published on an online cancer forum by users with cancer compared with family members or friends of those with cancer. Hence, responders to online cancer forums need to be cognizant of these differences in support needs and concerns and tailor their responses based on these findings.

## Introduction

### Background

Increasingly, individuals affected by cancer are seeking support on online cancer forums [1-4]. These forums function as a support group where individuals can seek and receive support around cancer from members of the forum, some of whom may (from their personal experience) be familiar with the support expressed.

Prior work determined that members of online cancer forums who self-declare to be diagnosed with cancer or going through cancer treatment tend to seek advice [5] and the more emotional support members of an online cancer forum received, the more likely they were to continue their membership in the forum [6].

The support needs and concerns expressed in online cancer forum posts may vary depending on who is accessing the forum; for example, the support needs expressed by individuals with cancer may vary from those of individuals who are family members or friends of individuals with cancer. In prior work, researchers have used language features from social media and online forum posts to determine whether users belong to different groups such as different age groups [7] and genders [8], to identify and characterize users who express loneliness from other users (who do not express loneliness) [9,10], and to predict patients risk for cardiovascular disease [11]. Similarly, in this paper, we analyze posts published on an online cancer forum on Reddit to determine the language features that delineate posts by users who self-declare to have or had cancer (we will refer to this group as the “has cancer” group) from posts by users who self-declare to be family members or friends of individuals with cancer (referred to as the “family or friend” group).

We hypothesize that these language features will reflect the differences in support needs and concerns expressed by users who belong to these different groups.

### Related Work

Users join online health forums to seek and give support as it relates to their health and well-being and that of others. Prior work has shown that online health forums are an effective way for seeking and giving support around mental health [12], substance use recovery [13,14], and cancer [1-4].

Prior work analyzed posts and comments on an online cancer forum and determined that members expressed more negative personal information in public messages compared with private messages [4] and the more emotional support members received, the higher the chance they will continue their membership in the forum [6]. Members of an online cancer forum who were either diagnosed with cancer or going through cancer treatment tended to seek advice and survivors of cancer shared their cancer-related experiences [5].

Over the course of their membership, members of an online cancer forum take on various roles on the forum and for individuals who have been members of the forum for a long period, these roles tend to be more focused on encouraging other members compared with their roles when they first became members of the forum, which tended to be related to seeking information [3]. These forums provide significant peer-to-peer support to individuals seeking support; hence, it is important that members of the forum responding to posts have an accurate understanding of the types of support being sought.

Our work in this paper is different from prior work analyzing posts in online cancer forums as they did not delineate posts by members of the forum that have/had cancer from those who are family or friends.

## Methods

### Data

Our data comprise posts from an active online cancer forum on Reddit, */r/Cancer*, which is the cancer forum with the most number of users (37,000 members as of March 2021) on Reddit. */r/Cancer* is self-described as “This reddit is for the discussion of cancer, cancer related news, stories of survival, stories of loss and everything else associated with the disease.” Using Google’s BigQuery [15], which is a data store with publicly available Reddit data sets, we collected 29,533 posts published between December 2015 and August 2019 on */r/Cancer*. From these posts, we identified users who self-declared to have or had cancer by selecting the user names of authors of posts that explicitly mentioned that the author of the post either has or had cancer; specifically, we selected posts which contained the word “cancer” and a first-person singular pronoun (ie, “I” and “me”), for example, “Just got diagnosed with lung cancer, how do I cope”. One of the coauthors (AA) reviewed these posts and took out the posts that were not indicating that a user has or had cancer. Similarly, we identified users who self-declared to be family members or friends of individuals with or that had cancer by selecting the user names of authors of posts that explicitly mentioned that a family member or friend has or had cancer; specifically, we selected posts which contained the word “cancer” and also contained the following keywords associated with family members and friends: “mother,” “mom,” “father,” “dad,” “parent,” “grand mother,” “grandmother,” “grand mom,” “grand ma,” “grand father,” “grandfather,” “grand dad,” “granddad,” “grand pa,” “husband,” “wife,” “spouse,” “son,” “daughter,” “child,” “aunty,” “aunt,” “uncle,” “nephew,” “niece,” “sister,” “brother,” “family,” “friend,” for example, “My young child is battling cancer.” One of the coauthors (AA) reviewed these posts and took out the posts that were not indicating that a user was a family member or friend of an individual with or that had cancer. Given the user names of users who either self-declared in posts to have or had cancer or were family members or friends of individuals with or that had cancer, we collected all their posts published in the forum (ie, */r/Cancer*). Table 1 shows a summary of our data set.

**Table 1:** Summary of our data set. This shows the number of posts by (1) users who self-declared to have or had cancer (the “has cancer” group) and (2) users who self-declared to be family members or friends (the “family or friend” group) of individuals with cancer.

Category	Number of posts	Number of users
The “has cancer” group	4414	2938
The “family or friend” group	3483	2456

### Differences in Language Use

We used 2 approaches to determine the differences in language use in posts by users who belong to either the “has cancer” group or the “family or friend” group. Specifically, we used (1) an open vocabulary method and (2) a dictionary-based method. In all the analysis in this work, we report the effect size by using Cohen *d*, which is the standardized difference between means.

### Open Vocabulary Method

In this section, we use a natural language processing topic modeling algorithm, latent Dirichlet allocation (LDA) [16], which is used to identify and group co-occurring words in documents (ie, Reddit posts in this work); these word groups are referred to as *topics*. LDA is a generative model which assumes that topics consist of a combination of words and tokens and Reddit posts consist of a mixture of topics. As words in Reddit posts are known, the latent variables of the topics can be estimated using Gibbs sampling [17]. Labels can be assigned to the various topics based on the content words associated with the topic. For example, LDA may cluster the words “Monday,” “Tuesday,” “Wednesday,” “Thursday,” and “Friday'” as days of the week. Using the DLATK package [18], we generated 20 LDA topics from the /r/Cancer posts by users that self-declared to have or had cancer (ie, the “has cancer” group) and users who self-declared to be family members or friends (ie, the “family or friend” group); we chose to generate 20 topics because we varied the number of LDA topics by using 10, 20, 30, and 40 topics, and one of the coauthors (AA) reviewed these topics and observed that the topic themes from 20 topics had the most coherent themes. Similar to prior works which used LDA to identify the topic themes from social media posts most associated with users who expressed loneliness from those who did not [9,10] and to delineate posts by individuals belonging to different age groups [7] and genders [8], we used the DLATK package [18] to identify the topic themes most associated with posts belonging to the “has cancer” group when compared with posts belonging to the “family or friend” group, and vice versa.

### Dictionary-Based Method

In this section, we used Linguistic Inquiry and Word Count (LIWC) [19], which is a psycholinguistic dictionary with 73 categories (eg, positive and negative emotions, health, and personal pronouns) and a curated list of words associated with these categories. Specifically, using the DLATK package [18], we determined the frequency of occurrence of words associated with LIWC categories in posts belonging to the “has cancer” group compared with the “family or friend” group.

### Ethics and Privacy

This study was deemed exempt by the Institutional Review Board guidelines of the authors institution. The data set used for this work is publicly available. The authors of this work did not contact any member or moderator of the forum /*r/Cancer* nor did we contact any Reddit users. Besides, Reddit user profile information was not reviewed or used in this work.

## Results

### Open Vocabulary Method

Table 2 shows the effect sizes (using Cohen *d*) of the most significant LDA topics (*P*<.001 [Benjamini–Hochberg *P* correction]) associated with */r/Cancer* posts by users that belong to the “has cancer” group compared with posts by users belonging to the “family or friend” group. In addition, Table 3 shows the effect sizes (using Cohen *d*) of the most significant LDA topics associated with */r/Cancer* posts by users belonging to the “family or friend” group compared with posts by users that belong to the “has cancer” group. The authors of the paper independently labeled each topic theme and then met to discuss and agree on the labels for each topic theme.

**Table 2:** LDA topics associated with posts by users who self-declared to have or had cancer (ie, the “has cancer” group) compared with posts by users in the “family or friend” group.

LDA^a^ topic themes	Highly correlated words in topics	Cohen *d*
Hospital visit	pain, hospital, back, days, blood, started, doctor, home, worse, ER	0.671
Questions/seeking advice	advice, good, wondering, experience, type, information, questions, survival, early, similar	0.537
Symptoms, risk, and cure of disease	cells, risk, cure, disease, symptoms, cancers, cervical, pancreatic, body, patients	0.474
Research/questions around cancer	research, patient, part, study, breast, questions, diagnosis, prostrate, find, survivor	0.432
Cancer surgery	surgery, colon, removed, tumor, thyroid, remove, lymph, kidney, nodes, stomach	0.349
Cost/payment for treatment	treatment, insurance, medical, money, health, clinical, working, options, pay, trials	0.345
Change in diet	eat, weight, food, stomach, throat, diet, healthy, tongue, taste, loss	0.293
Tests around cancer	scan, biopsy, back, doctor, results, CT, lymph, found, oncologist, tumor	0.290
Support from people/community	support, people, post, free, share, story, group, love, hope, great	0.245
Side effects of treatment	chemo, treatment, radiation, side, effects, week, hair, round, pretty, started	0.214

^a^LDA: latent Dirichlet allocation.

**Table 3:** LDA topics associated with posts by users who self-declared to be family members or friends of individuals with or that had cancer (ie, the “family or friend” group) compared with posts by users in the “has cancer” group.

LDA^a^ topic themes	Highly correlated words in topics	Cohen *d*
Losing family member	mom, day, passed, lost, home, didn't, love, hospital, wanted, made	0.702
Caring for family member	sister, brother, family, wife, home, work, parents, mother, live, care	0.373
Diagnosis of family member	dad, he’s, father, diagnosed, stage, ago, found, lung, today, pancreatic	0.339
Diagnosis of family member	mom, stage, breast, diagnosed, advice, she's, friend, ovarian, grandma, lung	0.179
Talk around support	time, life, family, things, make, support, care, health, long, difficult	0.159

^a^LDA: latent Dirichlet allocation.

### Dictionary-Based Method

Table 4 shows the effect sizes (using Cohen *d*) and LIWC categories that are more associated with posts belonging to the “has cancer” group when compared with the “family or friend” group. In addition, Table 5 shows the effect sizes (using Cohen *d*) and LIWC categories that are more associated with posts by the “family or friend” group when compared with posts by the “has cancer” group.

**Table 4:** LIWC categories most associated with posts belonging to the “has cancer” group when compared with the “family or friend” group. Effect size is reported as Cohen *d*.

LIWC^a^ category	Cohen *d*
Health	0.635
Biological processes	0.607
Second-person pronouns	0.234
Anxiety	0.126

^a^LIWC: Linguistic Inquiry and Word Count.

**Table 5:** LIWC categories most associated with posts belonging to the “family or friend” group when compared with posts by the “has cancer” group. Effect size is reported as Cohen *d*.

LIWC^a^ category	Cohen *d*
Third-person singular pronoun	1.168
Personal pronoun	0.977
Female references	0.964
Male references	0.746
First-person singular pronouns	0.543
Past focus	0.465
Affiliation	0.398
First-person plural pronouns	0.242
Sadness	0.224
Time	0.222
Present focus	0.221
Death	0.181
Friends	0.175

^a^LIWC: Linguistic Inquiry and Word Count.

## Discussion

### Principal Findings

In this work, using LDA and LIWC, we show that there are differences in the support needs and concerns expressed in online cancer forum posts by users who belong to the “has cancer” group compared with those belonging to the “family or friend” group. In the following section, we summarize the findings from this work.

In our analysis, we observed that users who self-declare to have or had cancer tend to post about topic themes such as their hospital visits and seeking advice and information as these relate to cancer; this finding is in line with previous work [5], which showed that individuals who self-declared (in an online cancer forum) to be diagnosed with cancer or undergoing treatment mostly sought advice from other members of the forum. We also observed that users who self-declared to have cancer tend to post about topics themes related to the cost/payments for their treatments, change in diet, and side effects of treatment, and use words associated with LIWC categories related to health and anxiety. These findings can aid in the design of processes for providing better support on online cancer forums. For example, the cost for cancer treatment can be expensive, and because users who self-declare to have or had cancer tend to post about topic themes related to cost/payment for their treatment, online cancer forums can partner with health care providers and relevant organizations to come up with and document detailed ways and tips in which patients with cancer can approach paying for their treatment; this information can be made easily available and accessible to users on the online forum. A similar thing can be done for other user concerns such as change in diet and side effects of treatments. Given that LIWC categories associated with anxiety are more associated with users who self-declared to have or had cancer, online cancer forums can provide/recommend professional mental health services to these users.

For users who self-declared to be family members or friends of individuals diagnosed with cancer, we observed that they tend to post about topic themes such as losing a family member, caring for a family member, and the diagnosis of a family member; also, these users tend to use words associated with LIWC categories focusing on the past/present, sadness, and death. Given that some of the topic themes users who self-declare to be family members or friends tend to post about are caring for a family member and the diagnosis of a family member, online cancer forums can partner with health care providers to document ways in which these users can provide support and care to their loved ones with cancer—this information can be made easily accessible on the forum. Besides, given that LIWC categories associated with past/present, sadness, and death are more associated with the “family or friends” group, this may imply that users belonging to this group express (in their posts) having a difficult time coping with either losing their loved one or their loved one being sick; hence, the cancer forum can provide professional mental health counselors who can provide help to these users on how to cope with a loved one being sick or losing a loved one.

### Limitation

Prior work determined that the interests of members of online forums focused on similar topics may differ [20]; hence, a limitation of this work is that the language used on */r/Cancer* may differ from that used in other online cancer forums. In addition, the sample used in this work is composed of Reddit users who publish posts on the subreddit */r/Cancer* and is not representative of all users affected by cancer.

### Conclusion

In this paper, using LDA and LIWC, we determined the LDA topics and LIWC categories associated with posts by (1) users who self-declared to have or had cancer and (2) users who self-declared to be family members or friends of individuals with cancer; also, we observed that these language use differences reflect the differences in support needs and concerns expressed in posts belonging to these groups.

## Abbreviations

LDA: latent Dirichlet allocation
LIWC: Linguistic Inquiry and Word Count
